# Association between MR-proADM concentration and treatment intensity of antihypertensive agents in chronic kidney disease patients with insufficient blood pressure control

**DOI:** 10.1038/s41598-021-01403-2

**Published:** 2021-11-09

**Authors:** Motoshi Iwao, Ryota Tanaka, Yosuke Suzuki, Takeshi Nakata, Kohei Aoki, Akihiro Fukuda, Naoya Fukunaga, Ryosuke Tatsuta, Keiko Ohno, Hirotaka Shibata, Hiroki Itoh

**Affiliations:** 1 Department of Clinical Pharmacy, Oita University Hospital, Hasama-machi, Oita, Japan; 2 Department of Medication Use Analysis and Clinical Research, Meiji Pharmaceutical University, Noshio, Tokyo, Japan; 3 Department of Endocrinology, Metabolism, Rheumatology and Nephrology, Faculty of Medicine, Oita University, Hasama-machi, Oita, Japan

**Keywords:** Biomarkers, Hypertension

## Abstract

Response to antihypertensive drugs in patients with chronic kidney disease (CKD) has great interindividual variability. Adrenomedullin (ADM) is produced abundantly in hypertension, but clearance is very rapid. Mid-regional proADM (MR-proADM) produced from an ADM precursor is considered a surrogate biomarker for quantification of ADM. We investigated the association of MR-proADM with antihypertensive resistance in CKD patients with poor blood pressure (BP) control. This cross-sectional study analyzed 33 CKD patients with poor BP control defined as failure to achieve target BP despite at least two classes of antihypertensive drugs. Treatment intensity score was calculated to facilitate comparability of antihypertensive regimens across subjects taking different drugs. Plasma MR-proADM concentration was measured using ultra-performance liquid chromatography coupled with tandem mass spectrometry. Plasma MR-proADM concentration correlated with estimated glomerular filtration rate (eGFR) (r =  − 0.777, *p* < 0.001). Treatment intensity score correlated positively with plasma MR-proADM concentration (r = 0.355, *p* = 0.043), and the correlation was further enhanced after correction by weight (r = 0.538, *p* = 0.001). Single and multiple regression analysis identified MR-proADM concentration (*p* = 0.005) as independently associated with weight-corrected treatment intensity score. MR-proADM may be useful as a biomarker to determine the therapeutic intensity of antihypertensive drugs in CKD patients with poor BP control.

## Introduction

Chronic kidney disease (CKD) is defined in the Kidney Disease: Improving Global Outcomes (KDIGO) guideline as abnormalities of kidney structure or function, present for more than 3 months, with implications for health^1^. Previous reports suggested that the progression of CKD is a risk factor for complication with cardiovascular disease (CVD)^2,3^. Preventing CKD progression decreases the risk of complications of several diseases, contributing to reducing the burden of medical economics and extending healthy life^2,3^.

Hypertension is a major risk factor for impaired renal function. In the United States, hypertension is the second leading cause of end-stage kidney disease (ESKD)^4^. On the other hand, many CKD patients are complicated with secondary hypertension due to renal parenchymal disorder^5^. CKD and hypertension are closely related pathophysiological states and exacerbate each other. Moreover, hypertension is also an independent risk factor for CVD. Appropriate management of blood pressure is important to prevent the development of CVD as well as the progression of CKD.

Many patients with hypertension receive antihypertensive therapy in addition to modification of lifestyle to achieve target blood pressure. However, some patients, especially those who have CKD, exhibit poor response to antihypertensive therapy, and determination of the optimal antihypertensive therapy to achieve the target blood pressure takes time^6^. In general, blood pressure is lowered slowly over several months after initiation of antihypertensive therapy. However, patients with high risk for CVD are recommended to achieve target blood pressure within a few weeks if possible, because the difference in blood pressure lowering during 1–3 months after antihypertensive initiation affects the onset of CVD^7^. Thus, to achieve target blood pressure as soon as possible, a biomarker that can predict the response of each patient to antihypertensive therapy before starting antihypertensive therapy is required.

Adrenomedullin (ADM), a bioactive peptide related to cardiovascular homeostasis, is reported to be a predictor of the development of various diseases such as hypertension^8^. ADM is produced from cells in various organs, especially vascular endothelial cells, and exerts some important effects to maintain vascular homeostasis. In the kidney, ADM is known to have renoprotective function by increasing renal blood flow^9^. Furthermore, ADM would regulate proadrenomedullin N-terminal 20 peptide (PAMP) secretion in the juxtaglomerular complex^10^. Since ADM and PAMP could regulate renin activity, these peptides potentially play a significant role in maintaining systemic blood pressure. Increased blood ADM concentration has been associated with the development of hypertension in normotensive subjects^11^. However, ADM is difficult to quantify in the clinical setting, because of its nonspecific metabolism in blood, clearance due to binding to receptors and binding to plasma proteins^12–14^. Mid-regional proADM (MR-proADM) is produced from an ADM precursor peptide in equal amount as ADM and has been anticipated to be an effective surrogate biomarker for quantification of ADM because of its low physiological activity and high stability in the body compared with ADM^15^. Focusing on the potential of MR-proADM as a cardiovascular biomarker, we previously reported a relationship between MR-proADM level and resistance to antihypertensive therapy in stable kidney transplant recipients^16^. Thus, we hypothesized that MR-proADM level serves as a predictive biomarker for the responsiveness to antihypertensive drugs in CKD patients with poor blood pressure control.

Given the above background, we conducted a cross-sectional study in CKD patients with poor blood pressure control and determined the relation between MR-proADM and antihypertensive resistance.

## Methods

### Patients

This cross-sectional study recruited CKD outpatients with poor blood pressure control, who attended the Department of Nephrology in Oita University Hospital. According to the Guidelines for the Management of Hypertension 2014 published by the Japanese Society of Hypertension^17^, poor blood pressure control was defined as failure to achieve the target systolic and diastolic blood pressure despite antihypertensive therapy with at least two different classes of drugs. Using conventional office blood pressure, the target blood pressure was determined according to the above guideline^17^. The target blood pressure was < 130/80 mmHg for CKD patients with proteinuria; < 140/90 mmHg for young, middle-aged, and older patients aged below 75 without proteinuria; < 150/90 mmHg for older patients aged 75 or over without proteinuria; < 130/80 mmHg for diabetic patient; and < 140/90 mmHg for patients with cerebrovascular disorder or coronary artery disease. CKD was defined as glomerular filtration rate (GFR) lower than 60 mL/min/1.73 m^2^, according to the Clinical practice guidebook for diagnosis and treatment of chronic kidney disease 2018 published by the Japanese Society of Nephrology^18^. Estimated GFR (eGFR) was calculated using the CKD Epidemiology Collaboration (CKD-EPI) equation and was substituted for GFR^19^. Exclusion criteria were as follows: patient under 30 or over 80 years of age; patient with a history of infectious diseases within 1 week before recruitment or acute heart failure within 1 month before recruitment; and patient undergoing dialysis.

### MR-proADM measurement

Blood sampling from recruited CKD outpatients was conducted at the time of registration. Blood sample was drawn from a vein into a tube containing ethylenediaminetetraacetic acid. After centrifugation (5 min at 2330×*g*, 25 °C), plasma sample was collected and frozen at − 40 °C until assay. The plasma MR-proADM concentrations were measured using an ultra-performance liquid chromatography coupled with tandem mass spectrometry (UPLC-MS/MS) method that we developed previously^20^. The UPLC–MS/MS assay has high specificity and sensitivity, a lower limit of quantification of 0.4 ng/mL and a calibration range of 0.4–100 ng/mL. Within-batch and batch-to-batch accuracy for three quality control samples ranged from −0.69 to 8.05% and from 1.72 to 5.76%, respectively. Within-batch and batch-to-batch precision ranged from 1.94 to 10.8% and from 7.17 to 8.15%, respectively.

### Clinical data and treatment intensity score

The following clinical data were collected: sex, age, body weight, systolic and diastolic blood pressure, prescribed drugs and primary diseases. The recorded laboratory data included serum creatinine, blood urea nitrogen and urine protein. Estimated quantitative proteinuria was calculated as urine protein to creatinine ratio. To evaluate the degree of treatment resistance in patients taking various doses of different antihypertensive drugs, the treatment intensity score was calculated as the sum of “daily dose/maximum daily dose” of all antihypertensive drugs used^21^. For example, treatment intensity score for a patient taking amlodipine 10 mg and telmisartan 40 mg is 1.5 (score for amlodipine (maximum daily dose 10 mg) = 10 mg/10 mg = 1; score for telmisartan (maximum daily dose 80 mg) = 40 mg/80 mg = 0.5). Analyses were conducted using both the treatment intensity score and the weight-corrected treatment intensity score.

### Statistical analysis

The relations of plasma MR-proADM concentration with clinical data and treatment intensity score with and without correction by weight were evaluated. The relation of plasma MR-proADM concentration with the weight-corrected treatment intensity score for each of the four drug classes (calcium-channel antagonists, ACE inhibitors and ARBs, β blockers and αβ blockers, and diuretics) were also evaluated. Since the patients in this analysis received antihypertensive therapy with at least two different classes of drugs, there was overlap of patients among the four groups; i.e., a patient in one drug class was also taking antihypertensive drug(s) of other class(es) (Supplementary Table 1). Correlation between variables was analyzed using Spearman’s rank correlation coefficient. Single and multiple regression analyses by stepwise selection was performed using treatment intensity score as the dependent variable. As covariates for the single and multiple regression analyses, sex, age, eGFR, plasma MR-proADM concentration, and body mass index were selected for the following reason: commonly, blood pressure increases with age and tends to be higher in men than women; eGFR is the indicator of renal function closely related to hypertension; MR-proADM and BMI were previously reported to be independently associated with treatment intensity score in stable kidney transplant recipients, in multiple regression analysis^16^. A *p*-value less than 0.05 was considered statistically significant. Statistical analyses were performed using the Predictive Analysis Software Statistics version 21.0 (SPSS Inc., IL, USA).

### Ethics policy

The study was conducted in accordance with the ethical standards of the our institute and with the Helsinki Declaration of 1975, as revised in 2013. The protocol for this study was approved by the Oita University Faculty of Medicine Ethics Committee (review reference number: 1005). All subjects provided written informed consent for participation in this study before the study started.

## Results

### Patient characteristic

Thirty-three CKD patients with poor blood pressure control were recruited. The clinical and laboratory data at registration are shown in Table 1. The drugs used for hypertension were mainly calcium channel blockers and angiotensin-converting enzyme inhibitors or angiotensin receptor blockers (Table 2). The (mean ± standard deviation) calculated treatment intensity score was 2.11 ± 1.07. The (mean ± standard deviation) eGFR was 31.0 ± 14.9 mL/min/1.73 m^2^ and measured plasma MR-proADM concentration was 2.99 ± 1.85 ng/mL. IgA nephropathy and nephrosclerosis were the main primary diseases of CKD.

**Table 1:** Baseline characteristics**.**

Characteristics	Value
Sex (male/female)	19/14
Age (years)	64.1 ± 12.3
Duration of hypertension (year)	7.96 ± 6.04
Systolic blood pressure (mmHg)	140.5 ± 13.2
Diastolic blood pressure (mmHg)	80.8 ± 11.0
Smoking history (%)	45.5%
Serum creatinine (mg/dL)	2.07 ± 1.01
Blood urea nitrogen (mg/dL)	35.1 ± 14.9
eGFR (mL/min/1.73m^2^)	31.0 ± 14.9
Urine protein (mg/dL)	118.0 ± 122.8
Estimated quantitative proteinuria (g/g Cr)^a^	1.62 ± 1.74
MR-proADM concentration (ng/mL)	2.99 ± 1.85
Treatment intensity score	2.11 ± 1.07
**Primary disease of CKD**	
IgA nephropathy	10
Nephrosclerosis	9
Diabetic nephropathy	3
Membranous nephropathy	3
Lupus erythematosus nephritis	2
Others	6
Medical history of diabetes	18

*eGFR* estimated glomerular filtration rate, *Cr* creatinine, *MR-proADM* mid-regional pro-adrenomedullin, *CKD* chronic kidney disease.

^a^Estimated quantitative proteinuria was calculated as urine protein to creatinine ratio.

**Table 2:** Prescribed antihypertensive drugs**.**

**Number of hypertensive drug classes used in combination**	
2	15 (45.5%)
3	16 (48.5%)
4	2 (6.0%)
**Antihypertensive drugs taken at the time of registration**	
Calcium-channel antagonists	30 (90.9%)
ACE inhibitors and ARBs	29 (87.9%)
β blockers and αβ blockers	12 (36.4%)
**Diuretics**	12 (36.4%)
Loop	9 (27.2%)
Potassium-sparing	3 (9.0%)
Thiazide	1 (3.0%)
Others	3 (9.0%)

Data are expressed as number of patients (percent).

*ACE* angiotensin converting enzyme, *ARB* angiotensin receptor blocker.

### Correlation of plasma MR-proADM concentration with eGFR and treatment intensity score

**Figure 1:**
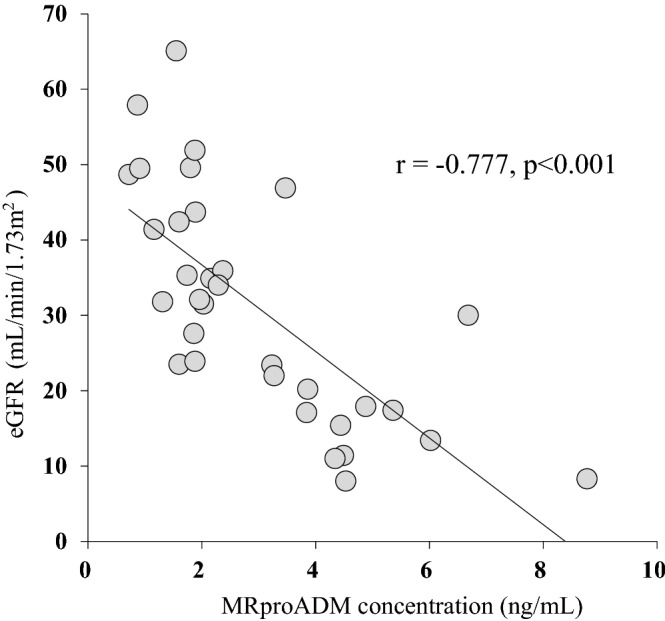
Correlation between mid-regional pro-adrenomedullin (MR-proADM) and estimated glomerular filtration rate (eGFR) in chronic kidney disease patients with poor blood pressure control.

**Figure 2:**
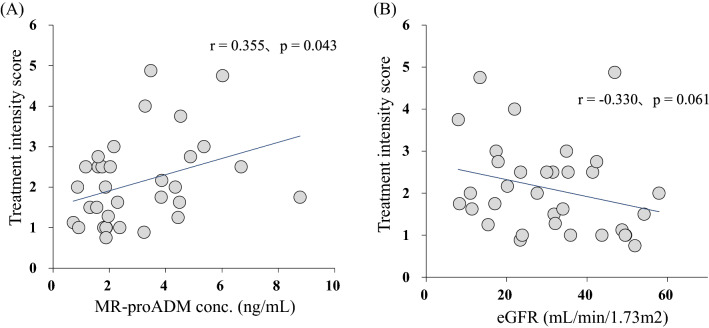
Correlation between treatment intensity score and **(A)** mid-regional pro-adrenomedullin (MR-proADM) concentration, **(B)** estimated glomerular filtration rate (eGFR) in chronic kidney disease patients with poor blood pressure control.

**Figure 3:**
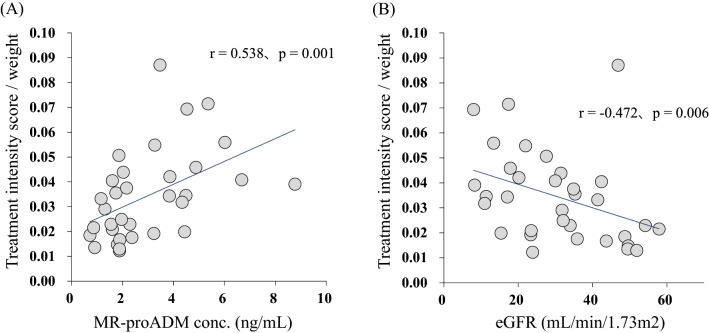
Correlation between weight-corrected treatment intensity score and **(A)** mid-regional pro-adrenomedullin (MR-proADM) concentration, **(B)** estimated glomerular filtration rate (eGFR) in chronic kidney disease patients with poor blood pressure control.

The relation between plasma MR-proADM concentration and eGFR is shown in Fig. 1. Plasma MR-proADM concentration correlated negatively with eGFR (r =  − 0.777, *p* < 0.001). The relations of treatment intensity score calculated for all the antihypertensive drugs taken by each patient with plasma MR-proADM concentrations and with eGFR are shown in Fig. 2. Treatment intensity score correlated positively with plasma MR-proADM concentration (r = 0.355, *p* = 0.043), but did not correlate with eGFR (r =  − 0.330, *p* = 0.061). On the other hand, the weight-corrected treatment intensity score showed a significant correlation with both eGFR (r =  − 0.472, *p* = 0.006) and plasma MR-pro ADM concentration (r = 0.538, *p* = 0.001) (Fig. 3). After correcting the treatment intensity score by weight, the correlation with plasma MR-pro ADM concentration was enhanced from r = 0.35 to r = 0.538. However, there was no significant correlation between plasma MR-proADM concentration and BMI and no significant difference in plasma MR-proADM concentration between overweight patients (BMI ≥ 25 kg/m^2^) and non-overweight patients (Supplementary Fig. 1).

**Figure 4:**
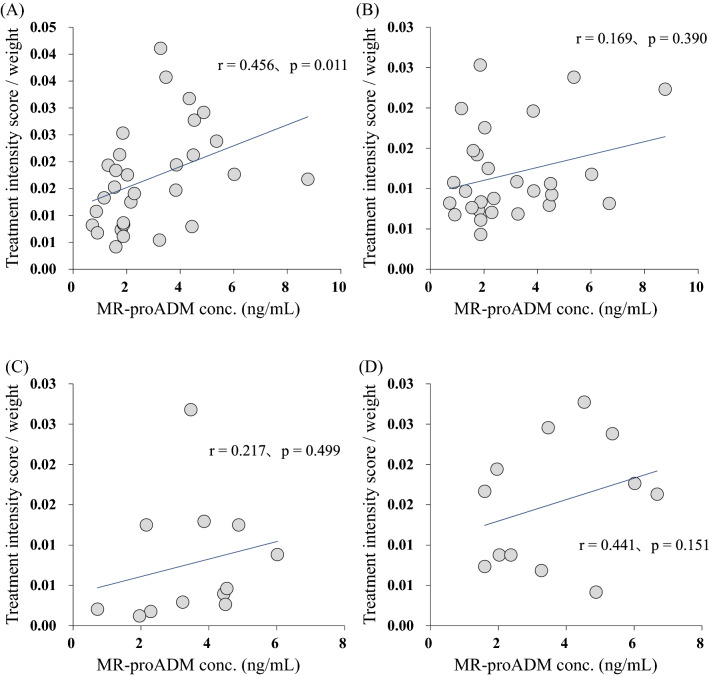
Correlation between mid-regional pro-adrenomedullin (MR-proADM) concentration and weight-corrected treatment intensity score calculated for each antihypertensive drug class: **(A)** calcium-channel antagonists, **(B)** ACE inhibitors and ARBs, **(C)** β blockers and αβ blockers, and **(D)** diuretics.

Next, we calculated the weight-corrected treatment intensity score for each antihypertensive drug class, and examined the relation of MR-proADM concentration with the weight-corrected treatment intensity score of each of the four drug classes (calcium-channel antagonists, ACE inhibitors and ARBs, β blockers and αβ blockers, and diuretics). No significant correlation was found for three drug classes (ACE inhibitors and ARBs, β blockers and αβ blockers, and diuretics), whereas the weight-corrected treatment intensity score for calcium-channel antagonists correlated positively with plasma MR-proADM concentration (r = 0.456, *p* = 0.011) (Fig. 4).

Single regression analysis was performed using weight-corrected treatment intensity score of all the drugs taken by each patient as the dependent variable and plasma MR-proADM concentration, sex, age, body mass index and eGFR as independent variables. Stepwise multiple regression analysis was performed using as independent variables those factors (plasma MR-proADM concentration, body mass index and eGFR) with *p*-values of 0.2 or less in single regression analysis. Multiple regression analysis identified only MR-proADM concentration (*p* = 0.005) as the independent factor associated with weight-corrected treatment intensity score (Table 3).

**Table 3:** Single and multiple regression analysis for factor associated with weight-corrected treatment intensity score.

Independent variable	Single regression analysis	Multiple regression analysis		
	*p*-value	*p*-value	Estimate	95% CI
Plasma MR-proADM concentration	0.005	0.005	0.0046	0.0015 to 0.0077
Sex	0.342			
Age	0.254			
Body mass index	0.075			
eGFR	0.033			

Total R^2^ for the model was 0.224.

*MR-proADM* mid-regional pro-adrenomedullin, *eGFR* estimated glomerular filtration rate, *95% CI* 95% confidence interval.

## Discussion

Patients with CKD often exhibit resistance to antihypertensive drug, and they require much time to attain the target blood pressure. As sustained hypertension is associated with the progression of CKD or the development of CVD, a biomarker that reliably determines the therapeutic intensity of antihypertensive drugs is useful for early achievement of the target blood pressure. We focused on MR-proADM as the biomarker and evaluated the relationship between plasma MR-proADM concentration and treatment intensity score in CKD patients with poor blood pressure control. This study revealed several findings: (1) eGFR correlated negatively with plasma MR-proADM concentration; (2) treatment intensity score of all drugs taken by each patient correlated positively with plasma MR-proADM concentration, but not with eGFR; (3) the correlation of treatment intensity score with plasma MR-proADM concentration was enhanced after correction by weight; (4) stepwise multiple regression analysis identified MR-proADM concentration as the only independent factor associated with weight-corrected treatment intensity score.

In this study, a negative correlation between eGFR and plasma MR-proADM concentration was found, suggesting that higher renal function correlates with lower plasma MR-proADM concentration. Dieplinger et al.^22^ reported that plasma MR-proADM concentration correlated strongly with GFR measured by iohexol, suggesting that MR-proADM is excreted mainly by filtration in the kidney, especially via the glomeruli. However, at present, the excretion routes of MR-proADM remain unclear, and the possibility of excretion by other routes cannot be ruled out. Renal damage per se may also influence plasma MR-proADM concentration. ADM would regulate PAMP secretion in the juxtaglomerular complex^10^. Since ADM and PAMP could regulate renin activity, these peptides potentially play a role in systemic blood pressure control and vascular remodeling. Furthermore, ADM is a bioactive peptide identified in sclerotic glomerular cells and fibroblasts in the kidney^23^ and is known to have renoprotective function by increasing renal blood flow^9^. Since ADM plays a central role in the compensatory mechanism of CKD, plasma ADM level has been considered to increase with renal dysfunction^24^. In a previous study^22^, MR-proADM concentration was identified as an independent factor predicting the progression of CKD even after baseline correction by GFR and proteinuria, suggesting that renal injury per se influences the fluctuation of MR-proADM concentration. Similarly, several studies have reported that plasma MR-proADM levels are high in patients with renal failure and increase as the CKD stage progresses^20,25,26^. These findings support the results in this study.

While plasma MR-proADM concentration correlated positively with treatment intensity score, eGFR did not correlate with treatment intensity score. It is commonly known that CKD and hypertension are physiologically related to each other. However, CKD patients with poor blood pressure control are at risk of developing arteriosclerosis, cardiac disease, and other illnesses due to long-term exposure to high blood pressure. Moreover, poor blood pressure control is caused by many factors such as poor adherence; lifestyle factors such as excessive salt intake, obesity and excessive drinking; sleep apnea syndrome; excessive fluid volume; and secondary hypertension^27^. For CKD patients with poor blood pressure control, various factors other than renal failure could cause hypertension and resistance to antihypertensive drugs. Therefore, our finding of no significant correlation between eGFR and treatment intensity score in CKD patients with poor blood pressure control in this study was as expected. On the other hand, the treatment intensity score correlated positively with plasma MR-proADM concentration. Moreover, stepwise multiple regression analysis identified plasma MR-proADM concentration as the only independent factor associated with the treatment intensity score. The increase in treatment intensity score, which signifies increased treatment resistance to antihypertensive drugs, is considered to be due to vascular failure caused by vascular injury associated with hypertension and renal injury. ADM is released from the blood vessel wall and possesses blood pressure lowering activity^28^. Wild et al.^29^ showed elevated plasma MR-proADM concentration in patients with hypertension. Furthermore, Koyama et al.^30^ revealed a significant association of plasma MR-proADM concentration with brachial-ankle pulse wave velocity, which is an indicator of arterial stiffness, and suggested that MR-proADM is more suitable for diagnosing arterial stiffness as the criterion for vascular failure. Given these lines of evidence, MR-proADM concentration reflects the vascular condition in hypertensive patients, and its plasma concentration becomes higher under conditions of increased treatment resistance to antihypertensive drugs. Hence, the treatment intensity score correlates with MR-proADM concentration, unlike eGFR.

The correlation between treatment intensity score and plasma MR-proADM concentration was enhanced after treatment intensity score was corrected by weight. In general, heavy patients tend to receive higher doses of antihypertensive drugs since drug clearance and distribution volume are proportional to weight. This probably accounts for the improved correlation between weight-corrected treatment intensity score and plasma MR-proADM concentration. On the other hand, although eGFR also correlated significantly with weight-corrected treatment intensity score, eGFR was not identified as an independent factor in stepwise multiple regression analysis, unlike plasma MR-proADM concentration. A large correlation coefficient was found between eGFR and plasma MR-proADM concentration (r =  − 0.777). Therefore, we speculate that the significant correlation between weight-corrected treatment intensity score and eGFR is due to the indirect influence of the increased correlation of treatment intensity score with plasma MR-proADM concentration. However, since there are no reports of the association between MR-proADM concentration and the treatment intensity score in non-CKD patients with hypertension, the exact reason remains unclear.

There was no correlation between plasma MR-proADM concentration and weight-corrected treatment intensity score for three drug classes (ACE inhibitors and ARBs, β blockers and αβ blockers, and diuretics), but a positive correlation was found only for calcium-channel antagonists. Calcium channel blockers dilate arteries and effectively lower blood pressure by reducing calcium flux into vascular smooth muscle cells^31^. Since these drugs act directly on blood vessels and MR-proADM concentration reflects the vascular condition, these may explain the correlation between treatment intensity score for calcium-channel antagonists and plasma MR-proADM concentration. However, ARB and some αβ blockers (carvedilol and arotinolol) exert antihypertensive effect by antagonizing angiotensin II receptor and α1-receptor, respectively, on vascular endothelial cells. Thus, the reason why plasma MR-proADM concentration correlates with treatment intensity score only for calcium channel blockers is unknown.

Our previous study in stable kidney transplant recipients also showed a correlation between plasma MR-proADM concentration and anti-hypertensive treatment score^14^. The present study differs from our previous study in several aspects; namely, study subject (chronic renal failure patients before transplantation versus stable renal transplant recipients) and blood pressure control poor (target blood pressure not achieved despite at least two classes of antihypertensive drugs) versus good (systolic and diastolic blood pressure controlled below 130 and 80 mmHg, respectively). The possible causes of hypertension in stable kidney transplant recipients include not only renal failure but also various factors such as excessive salt intake following rapid recovery of renal function, graft renal parenchymal disorder, adverse effects of immunosuppressive drugs, and angiopathy due to transplanted renal artery stenosis^32^. Therefore, the cause of hypertension in stable kidney transplant recipients is partially different from that in CKD patients before transplantation. In our previous report^14^, the correlation between treatment intensity score and MR-proADM was confirmed in patients with good blood pressure control, but it was unclear whether the correlation would be found in patients with poor blood pressure control. The present study demonstrated the relationship between MR-proADM and treatment intensity score in hypertensive CKD patients with poor blood pressure control as well, suggesting the usefulness of MR-proADM for predicting antihypertensive treatment resistance in hypertensive patients.

The present study has several limitations. First, the number of recruited CKD patients with poor blood pressure control was small (N = 33). Large-scale, prospective research will need to be conducted in the future. Second, some patients included in this study could have falsely high blood pressure reading. Since patient recruitment was based on conventional office blood pressure, patients with white coat hypertension could have been included. Moreover, adherence of these patients is also unknown. Third, we speculate that plasma MR-proADM level is related to antihypertensive drug tolerance because plasma MR-proADM level reflects the status of vascular insufficiency. However, we did not evaluate indicators of arterial stiffness, such as pulse wave velocity and ankle-brachial pressure index. Thus, the detailed mechanism of this relationship remains unknown. Fourth, treatment intensity score is a parameter indicating the therapeutic intensity of an antihypertensive drug. There is a possibility that the score may not adequately reflect resistance to antihypertensive treatment. Fifth, we evaluated the relation of plasma MR-proADM concentration with the weight-corrected treatment intensity score of each of four antihypertensive drug classes. However, since the patients in this analysis received antihypertensive therapy with at least two different classes of drugs, the possible influence of polytherapy on the result of this analysis cannot be ruled out.

In conclusion, this study suggests the usefulness of plasma MR-proADM concentration as a biomarker reflecting resistance to antihypertensive therapy in CKD patients with poor blood pressure control. For hypertensive patients at high risk of CVD, while early attainment of the target blood pressure is desirable to prevent onset of CVD, some patients with severe hypertension may need high-intensity antihypertensive therapy from the initiation of treatment, with a concern of adverse events. For these patients, safe and individualized antihypertensive therapy may be planned by selecting drug(s) based on the evaluation of resistance to antihypertensive drug by MR-proADM in advance.

## Supplementary Information

Supplementary Information.

## Data Availability

All data generated or analyzed during this study are included in this published article.
