# Supplementary material for: Association between MR-proADM concentration and treatment intensity of antihypertensive agents in chronic kidney disease patients with insufficient blood pressure control
Source: Sci Rep. 2021 Nov 9;11:21931. doi: 10.1038/s41598-021-01403-2 (PMC8578546; doi:10.1038/s41598-021-01403-2)
Supplement: Supplementary file 1 — Supplementary Information. [file 41598_2021_1403_MOESM1_ESM.docx]

**SUPPLEMENTARY MATERIALS**

|  | Calcium-channel antagonists | ACE inhibitors and ARBs | β blockers and  αβ blockers | Diuretics | Others |
| --- | --- | --- | --- | --- | --- |
| Patient-1 | 1 | 1 | 0 | 0 | 0 |
| Patient-2 | 1 | 1 | 1 | 0 | 0 |
| Patient-3 | 1 | 1 | 1 | 0 | 0 |
| Patient-4 | 1 | 1 | 1 | 0 | 0 |
| Patient-5 | 1 | 0 | 0 | 2 | 0 |
| Patient-6 | 2 | 1 | 0 | 0 | 1 |
| Patient-7 | 1 | 1 | 0 | 2 | 0 |
| Patient-8 | 1 | 1 | 0 | 0 | 0 |
| Patient-9 | 1 | 1 | 1 | 0 | 0 |
| Patient-10 | 0 | 1 | 0 | 1 | 1 |
| Patient-11 | 2 | 1 | 0 | 0 | 0 |
| Patient-12 | 2 | 1 | 1 | 0 | 0 |
| Patient-13 | 1 | 1 | 0 | 0 | 0 |
| Patient-14 | 2 | 1 | 1 | 2 | 0 |
| Patient-15 | 1 | 1 | 0 | 0 | 0 |
| Patient-16 | 1 | 1 | 1 | 0 | 0 |
| Patient-17 | 1 | 1 | 0 | 1 | 0 |
| Patient-18 | 2 | 0 | 2 | 1 | 0 |
| Patient-19 | 2 | 0 | 1 | 2 | 0 |
| Patient-20 | 2 | 1 | 2 | 2 | 0 |
| Patient-21 | 3 | 1 | 0 | 1 | 0 |
| Patient-22 | 1 | 1 | 0 | 0 | 0 |
| Patient-23 | 0 | 1 | 0 | 1 | 0 |
| Patient-24 | 1 | 1 | 1 | 0 | 0 |
| Patient-25 | 1 | 1 | 0 | 0 | 0 |
| Patient-26 | 1 | 2 | 0 | 0 | 0 |
| Patient-27 | 1 | 1 | 0 | 0 | 0 |
| Patient-28 | 1 | 1 | 0 | 0 | 0 |
| Patient-29 | 2 | 1 | 0 | 1 | 0 |
| Patient-30 | 1 | 1 | 0 | 0 | 0 |
| Patient-31 | 1 | 1 | 0 | 0 | 0 |
| Patient-32 | 1 | 1 | 0 | 0 | 0 |
| Patient-33 | 0 | 0 | 1 | 1 | 1 |

**Supplementary Table 1** The number of antihypertensive drugs of each class taken by each patient at the time of recruitment. ACE: angiotensin converting enzyme, ARB: angiotensin receptor blocker

**Supplementary Fig. 1** Correlation between plasma mid-regional pro-adrenomedullin (MR-proADM) concentration and body mass index (A) and comparison between non-overweight (BMI < 25) group and overweight (BMI ≥ 25) group (B).
